# Diffusion MRI tracks cortical microstructural changes during the early stages of Alzheimer’s disease

**DOI:** 10.1093/brain/awad428

**Published:** 2023-12-21

**Authors:** Nicola Spotorno, Olof Strandberg, Erik Stomrud, Shorena Janelidze, Kaj Blennow, Markus Nilsson, Danielle van Westen, Oskar Hansson

**Affiliations:** Clinical Memory Research Unit, Department of Clinical Sciences, Malmö, Lund University, 223 62 Lund, Sweden; Clinical Memory Research Unit, Department of Clinical Sciences, Malmö, Lund University, 223 62 Lund, Sweden; Clinical Memory Research Unit, Department of Clinical Sciences, Malmö, Lund University, 223 62 Lund, Sweden; Memory Clinic, Skåne University Hospital, 214 28 Malmö, Sweden; Clinical Memory Research Unit, Department of Clinical Sciences, Malmö, Lund University, 223 62 Lund, Sweden; Department of Psychiatry and Neurochemistry, Institute of Neuroscience and Physiology, The Sahlgrenska Academy, University of Gothenburg, 405 30 Gothenburg, Sweden; Clinical Neurochemistry Laboratory, Sahlgrenska University Hospital, 431 80 Mölndal, Sweden; Diagnostic Radiology, Institution for Clinical Sciences, Lund University, 221 85 Lund, Sweden; Clinical Memory Research Unit, Department of Clinical Sciences, Malmö, Lund University, 223 62 Lund, Sweden; Diagnostic Radiology, Institution for Clinical Sciences, Lund University, 221 85 Lund, Sweden; Clinical Memory Research Unit, Department of Clinical Sciences, Malmö, Lund University, 223 62 Lund, Sweden; Memory Clinic, Skåne University Hospital, 214 28 Malmö, Sweden

**Keywords:** cortical mean diffusivity, astrocytes, amyloid-β, Alzheimer’s disease, clinical trials

## Abstract

There is increased interest in developing markers reflecting microstructural changes that could serve as outcome measures in clinical trials. This is especially important after unexpected results in trials evaluating disease-modifying therapies targeting amyloid-β (Aβ), where morphological metrics from MRI showed increased volume loss despite promising clinical treatment effects.

In this study, changes over time in cortical mean diffusivity, derived using diffusion tensor imaging, were investigated in a large cohort (*n* = 424) of non-demented participants from the Swedish BioFINDER study. Participants were stratified following the Aβ/tau (AT) framework. The results revealed a widespread increase in mean diffusivity over time, including both temporal and parietal cortical regions, in Aβ-positive but still tau-negative individuals. These increases were steeper in Aβ-positive and tau-positive individuals and robust to the inclusion of cortical thickness in the model. A steeper increase in mean diffusivity was also associated with both changes over time in fluid markers reflecting astrocytic activity (i.e. plasma level of glial fibrillary acidic protein and CSF levels of YKL-40) and worsening of cognitive performance (all *P* < 0.01).

By tracking cortical microstructural changes over time and possibly reflecting variations related to the astrocytic response, cortical mean diffusivity emerges as a promising marker for tracking treatments-induced microstructural changes in clinical trials.

## Teaser

Spotorno *et al*. show that cortical mean diffusivity, a measure of cortical microstructure derived from diffusion MRI, is able to track disease progression on the Alzheimer’s disease continuum and could thus be a promising outcome measure for use in clinical trials.

**See Vieitez *et al.* (https://doi.org/10.1093/brain/awae054) for a scientific commentary on this article**.

## Introduction

A key component of clinical trials evaluating novel disease-modifying therapies for neurodegenerative diseases like Alzheimer’s disease (AD) is sensitive markers of downstream pathological events, including neuroinflammation, synaptic degeneration and loss of neurons. Morphological metrics like brain and hippocampal volume are established measures of atrophy that have already been included as outcome measures in several anti-amyloid-β (Aβ) clinical trials.^1-5^ However, such metrics provided puzzling results showing increased loss of brain volume despite evidence of target engagement and slowing of cognitive decline.^1,2,5^ These counter-intuitive results might be due to microstructural changes, reflecting, for example, changes in the neuroinflammatory response or fluid shift between the intra and extra cellular or intravascular space.^6,7^ In light of these results, in recent years there has been an increased interest in investigating whether monitoring changes in cortical microstructural with diffusion MRI (dMRI) could provide more suitable outcome measures for clinical trials. Previous investigations have shown differences in dMRI metrics across clinical groups^8-11^ and even significant correlations with markers of both Aβ and tau aggregation,^12,13^ and glial activity,^13-15^ suggesting dMRI might provide metrics sensitive to the changes in glial response induced by disease modifying therapies.^13^ However, these results are based on cross-sectional dMRI data or small cohorts; therefore, they do not provide clear evidence supporting the ability of dMRI-derived metrics to track changes over time, which is a critical need in trials evaluating novel drugs.

In this study, we investigated the changes over time in one of the most commonly used dMRI metrics for assessing diffusion rate in the cortex, namely mean diffusivity (MD), in a large cohort of individuals with either preclinical or prodromal AD stratified following the Aβ/tau (AT) framework.^16^ We evaluated associations between longitudinal changes in cortical MD and longitudinal changes in both CSF and plasma markers of astrocytic and microglial activity, such as GFAP, YKL-40 and soluble TREM2 (s-TREM2), in AD-biomarker-defined groups. To further test the clinical relevance changes in cortical MD, the association with longitudinal changes in cognitive performance was also tested. The overarching goal of the study is to investigate whether metrics derived from dMRI could provide potential outcome measures for clinical trials.

## Materials and methods

### Participants

Non-demented participants (*n* = 485) from the Swedish BioFINDER study (clinical trial ID: NCT01208675 inclusion/exclusion criteria as previously reported^17^) with multiple diffusion MRI assessment, and aged >60 years, were included in the study cohort. To capture the entire spectrum of early AD development from subthreshold Aβ levels to abnormal Aβ levels and cognitive symptoms, cognitively unimpaired participants with and without evidence of Aβ pathology and patients with mild cognitive impairment (MCI) with evidence of Aβ pathology at baseline were included. Quality control procedures on the diffusion scans, based on visual assessment of raw and processed data, blinded to clinical diagnosis, led to the exclusion of 61 participants due to poor data quality. Therefore, 424 participants who underwent at least one baseline and one follow-up scan [number of scans (range): 2–5, median = 3; follow-up years (range): 2–8, median = 4] were included in the final cohort. Participants were stratified as Aβ-negative/tau-negative, Aβ-positive/tau-negative and Aβ-positive/tau-positive based on CSF Aβ_42/40_ and CSF p-tau181 levels. Demographic and clinical characteristics are summarized in Table 1. All subjects gave written informed consent according to the Declaration of Helsinki, and the study was approved by the Ethical Review Board of Lund, Sweden.

**Table 1:** Demographic summary of the study cohort

	Aβ-negative/tau-negative	Aβ-positive/tau-negative	Aβ-positive/tau-positive
*n* (% female)	238 (64%)	100 (57%)	86 (50%)
Age	72 (5)	73 (5)	73 (5)
Years of education	13 (3)	12 (4)^c^	12 (3)^c^
*APOE* ɛ4 (%)	45 (19%)	64 (64%)^c^	60 (70%)^c^
MMSE	29 (1)	28 (2)^c^	27 (2) ^c^
mPACC	0.0 (0.6)	−0.8 (1.1)^c^	−1.4 (1.2)^c^
Trail Making Test-A^a^	45 (16)	53 (25)^c^	58 (30)^c^
Trail Making Test-B^a^	91 (34)	119 (62)^c^	111 (35)^c^
ADAS delayed recall	2 (2)	4 (3)^c^	6 (3)^c^
CSF Aβ_42_/Aβ_40_	0.096 (0.015)	0.049 (0.011)^c^	0.038 (0.009)^c^
CSF p-tau181^b^	16 (4)	20 (5)^c^	38 (9)^c^

Values are given as mean (standard deviation). Aβ-positive/negative defined based on a CSF Aβ_42_/Aβ_40_ of less than 0.066.^17^ Tau positivity was defined as 2 standard deviations from the mean p-tau181 level of the cognitively unimpaired Aβ-negative participants. Aβ = amyloid-β; ADAS = Alzheimer’s Disease Assessment Scale; MMSE = Mini-Mental State Examination; mPACC = modified version of the Preclinical Alzheimer Cognitive Composite.

^a^The scores for the Trail Making Tests are reported in seconds.

^b^CSF p-tau181 levels are reported in pg/ml. Both cognitive and biomarkers values refer to baseline measures.

^c^Significantly different from the Aβ-negative/tau-negative (*P* < 0.05).

### Imaging protocol and analysis

#### MRI protocol

MRI scans were performed on a Siemens Tim Trio 3 T scanner (Siemens Healthcare) equipped with a standard 12 channel head coil. A single-shot echo-planar imaging sequence was used to acquire 65 volumes [repetition time (TR)/echo time (TE): 8200/86 ms; resolution: 2 × 2 × 2 mm^3^; two b-values: 0 and 1000 s/mm^2^ distributed over 1 and 64 directions]. A T1-weighted magnetization-prepared rapid gradient-echo (MPRAGE) sequence was also acquired (TR/TE: 1950/3.4 ms, resolution: 1 × 1 × 1.2 mm^3^).

#### MRI processing

##### Processing of structural data

MPRAGE images were preprocessed using FreeSurfer longitudinal pipeline (version 6.0, https://surfer.nmr.mgh.harvard.edu). FreeSurfer pipeline includes several steps such as correction for intensity homogeneitysle,^18^ removal of non-brain tissue,^19^ segmentation into grey and white matter^20,21^ and estimation of cortical thickness.^22^ Critically, the longitudinal pipeline entails the creation of within subject templates as a first step of segmentation and reconstruction. Moreover, each time point is initialized within the template to reduce the variability in the optimization process.

##### Processing of diffusion-weighted images

The dMRI data were processed by using a combination of open-source algorithms. See the Supplementary material and Supplementary Fig. 1 for a more detailed description of the processing steps. In brief, the processing steps included denoising the dMRI series, removal of Gibbs ringing artifacts (both using MRtrix3^23^ routines) and correction of movement-induced artefacts and eddy currents using FSL Eddy (FMRIB Software Library, version 6.0.4; Oxford, UK) and partial correction of susceptibility induced distortion using a non-linear registration to the T1-weighted image using Advanced Normalization Tools (ANTs) routines (v2.1). The target of the registration was the T1-weighted image acquired in the same session as the dMRI series. The diffusion tensor model was fit using FSL tools and cortical MD was projected on the surface using a combination of ANTs and FreeSurfer routines.^13^ Both MD and cortical thickness values were extracted from the 68 cortical regions of the Desikan–Killiany atlas included in FreeSurfer. The atlas was project in the individual subject spaces following the steps of the FreeSurfer longitudinal pipeline to ensure both between- and within-subject consistency in the estimation of the metrics used in the statistical analyses. See the Supplementary material and Supplementary Fig. 1 for a more detailed explanation of the processing steps.

### CSF and plasma protocol and analysis

Both CSF Aβ_42_/Aβ_40_ and CSF p-tau181 were measured using the Roche Elecsys assay. Aβ-positive status was defined as a CSF Aβ_42_/Aβ_40_ ratio of less than 0.066.^17^ Tau positivity was defined as 2 standard deviations (SD) from the mean p-tau181 level of the cognitively unimpaired Aβ-negative participants (cut-off = 25.9 pg/ml). Longitudinal measurement of plasma levels of glial fibrillary filament protein (GFAP) and neurofilament light (NfL) were available for 322 participants (at least one baseline and one follow-up sample; mean follow-ups: 2.8, SD: 0.8, range: 2–4) and longitudinal CSF levels of YKL-40, GFAP and s-TREM2 were available for 292 participants (at least one baseline and one follow-up sample; mean follow-ups: 2.7, SD: 0.7, range: 2–4). The analysis was performed by board certified laboratory technicians, who were blinded to clinical diagnoses, using previously reported procedures^17^ and employing the Roche NeuroToolKit.^24,25^

### Cognitive tests

Global cognition was assessed using a modified version of Preclinical Alzheimer Cognitive Composite^26^ [mPACC; composed of the Alzheimer’s Disease Assessment Scale (ADAS) delayed recall test (*2), animal fluency, Mini-Mental State Examination (MMSE) and Trail Making-A; available for 373 participants, mean follow-ups: 3.0, SD: 0.9, range: 2–5). Executive functions were assessed with the Trail Making Test (TMT, focusing on part-B, available for 311 participants, mean follow-ups: 3, SD:1, range: 2–5) and memory function was probed with the ADAS delayed recall score (available for 418 participants, mean follow-ups: 3.1, SD: 0.9, range: 2–5).

### Statistical analysis

The relationships between demographic variables and clinical status (i.e. diagnosis) were evaluated with chi-square, ANOVA and Student’s *t*-test (Table 1).

Regional values of both cortical diffusion metrics and cortical thickness from the 68 region parcels of the Desikan–Killiany atlas were averaged across hemispheres leading to 34 regions of interest that were used in the analyses. Two *a priori* meta-regions of interest (ROIs) were also defined as previously reported^13,27,28^ focusing on region accumulating Aβ early in the disease process (early-Aβ ROIs) and on temporal neocortical regions (temporal ROIs). For each ROI, longitudinal changes in MD and cortical thickness were estimated by fitting a linear mixed-effect model (LME) including both random intercept and random slope (e.g. MD_ROI_ ∼ time; random = 1 + time | ID-participant). Individual-specific random slopes (i.e. the slopes estimated by the LME as random effects) were employed as proxy of longitudinal changes over time in the subsequent analyses. Changes over time in both fluid markers and cognitive measures were also estimated using individual-specific random slopes from LME. This strategy as already been employed in previous works.^29^ It is important to note that the estimation of the changes over time of imaging metrics and fluid or cognitive measures took place independently from one another. In other words, different LME models were fitted for each metrics and the same participant could have a different number of follow-ups dMRI scan and e.g. plasma samples available. A sensitivity analysis was also performed by accounting for changes over time in cortical thickness before performing the statistical analyses. To this aim, regional MD values were corrected for regional cortical thickness values using the covariance method.^30^ The residualized MD values were then used as input to LME models to derive cortical thickness-corrected (CT-corrected) estimates of changes over time in MD (see Supplementary material, ‘Results’ section and Supplementary Fig. 2 for further details).

#### Differences in cortical MD trajectories across groups

Multiple linear regression models were employed to investigate the differences in longitudinal cortical MD across groups in each region of interest. All models included age and sex as covariates (e.g. longitudinal-MD_ROI_ ∼ Biomarker-defined group + age + sex; longitudinal-MD_ROI_ = individual-specific random slopes from LME extracted from a specific ROI). In regions where the analysis revealed a significant association, a follow-up analysis was performed including baseline cortical thickness in the model as a proxy of neurodegenerative stage. A further sensitivity analysis was performed by restricting the comparison between the Aβ-negative/tau-negative group and the Aβ-positive/tau-negative group to participants who did not develop cognitive symptoms over the course of the follow-ups. Group differences in the meta-ROIs were also assessed using LME.

#### Associations between cortical MD trajectories and longitudinal changes in fluid markers

The association between longitudinal changes in regional MD and longitudinal changes in plasma levels of both GFAP and NfL as well as in CSF levels of GFAP, YKL-40 and s-TREM2 were tested in each ROI in a subset of participants with available samples using multiple linear regression models (e.g. longitudinal-MD_ROI_ ∼ longitudinal-GFAP + baseline-age + sex; longitudinal-MD_ROI_ = individual-specific random slopes from LME extracted from a specific ROI). In regions where the analysis revealed a significant association, a follow-up analysis including baseline cortical thickness in the model was performed.

The statistical significance of all region-wise analyses was set to the false discovery rate threshold of 0.05, employing the Benjamini–Hochberg procedure. Complementary analyses focusing on the two meta-ROIs was also performed (e.g. longitudinal-MD_meta-ROI_ ∼ longitudinal-GFAP + baseline-age + sex).

#### Associations between cortical MD trajectories and longitudinal changes in cognitive performance

The clinical relevance of longitudinal changes in cortical MD was further tested by investigating the associations of this metric with longitudinal changes in executive, memory functions and in global cognition. Changes over time in scores of cognitive testes were included as dependent variable in multiple regression models with longitudinal-cortical MD from one of the two meta-ROIs as independent variable along with age, sex and years of education (e.g. longitudinal-mPACC ∼ longitudinal-MD_meta-ROI_ + baseline-age + sex + education). All the analyses were performed in Python 3.7.6 and R v4.2.2.

## Results

### Regional changes over time in cortical MD differs between biomarker-defined groups

**Figure 1:**
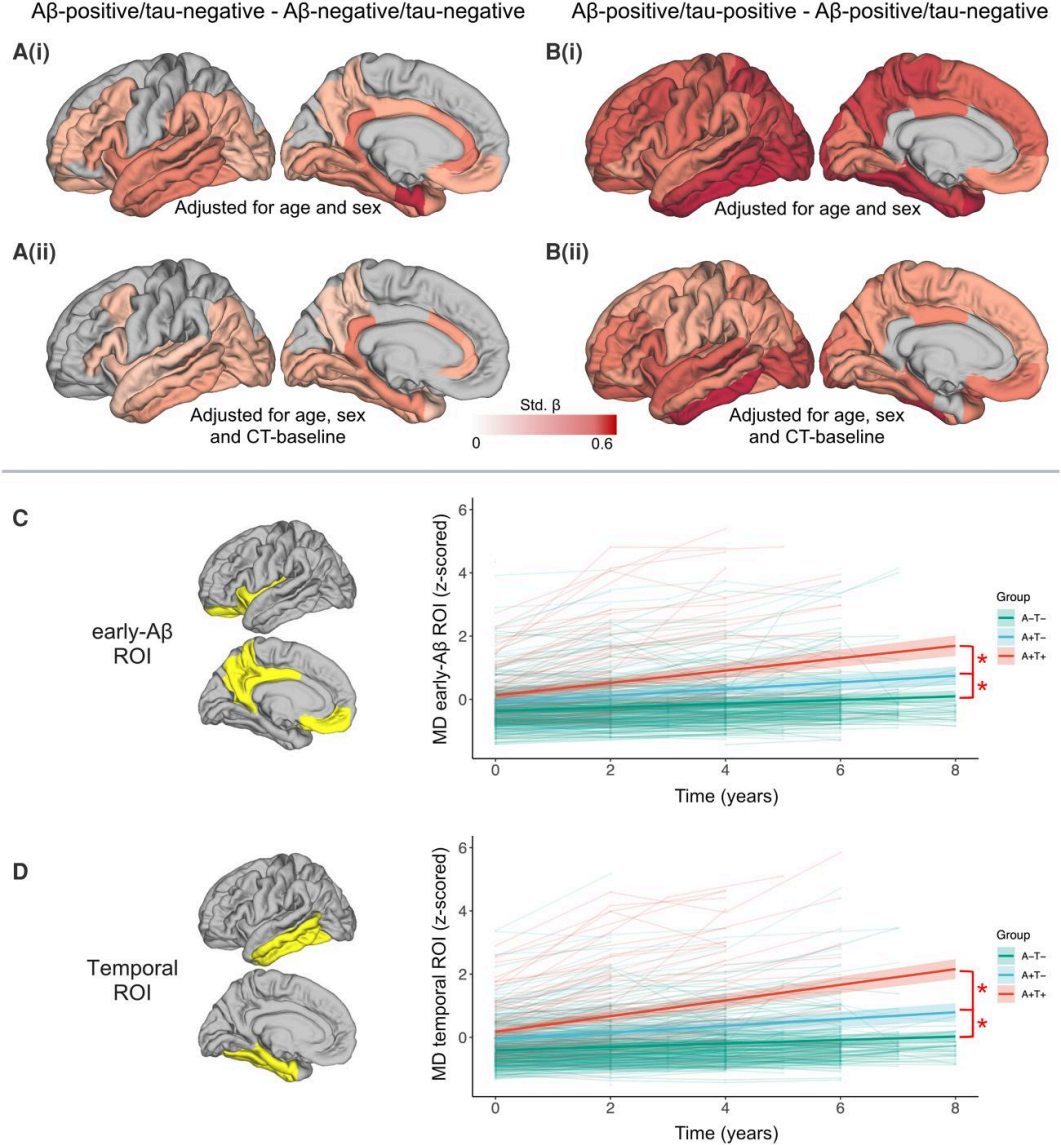
**Changes over time in cortical mean diffusivity across groups.** (**A** and **B**) The colour scale represents standardized-β values from the multiple regression model comparing mean diffusivity (MD) across groups. For visualization purposes, the standardized-β values of each region of interest have been plotted on a standard cortical surface. Only results corrected for multiple comparisons are displayed (false discovery rate, *P* < 0.05). Changes over time are defined by individualized slopes from the linear mixed-effect (LME) model. [**A**(**i**)] Difference in in cortical MD between amyloid-β (Aβ)-negative/tau-negative and Aβ-positive/tau-negative participants. [**A**(**ii**)] Difference in changes over time in cortical MD between Aβ-negative/tau-negative and Aβ-positive/tau-negative participants including baseline cortical thickness from the same region as a covariate in the model. [**B**(**i**)] Difference in changes over time in cortical MD between Aβ-positive/tau-negative and Aβ-positive/tau-positive participants. [**B**(**ii**)] Difference in changes over time in cortical MD between Aβ-positive/tau-negative and Aβ-positive/tau-positive participants including baseline cortical thickness from the same region as a covariate in the model. (**C** and **D**) Difference in changes over time in cortical MD focusing on two *a priori* defined meta-ROIs: one encompassing regions accumulating Aβ early in the disease process (**C**), one encompassing neocortical temporal regions (**D**). The spaghetti plots represent the individual trajectories while the regression lines report the results of the LME models described in the text. The shadowed areas around the regression line represent the 95% confidence interval. Both the data-points and the regression lines are colour-coded accordingly to the biomarker-defined groups (A−T− = Aβ-negative/tau-negative; A+T− = Aβ-positive/tau-negative; A+T+ = Aβ-positive/tau-positive).

Longitudinal cortical MD revealed widespread microstructural differences, namely a steeper increase in MD over time, in the Aβ-positive/tau-negative group when compared with the control (Aβ-negative/tau-negative) group, with higher standardized-β coefficients in neocortical temporal regions, as well as rostral anterior and posterior cingulate cortex [standardized-β range: 0.21–0.59; Fig. 1A(i)]. When including baseline cortical thickness of the same region as a proxy of neurodegenerative stage the results remain largely unchanged [standardized-β range: 0.16–0.42; Fig. 1A(ii)]. After removing the participants who developed cognitive symptoms over the course of the follow-ups, the comparison between the Aβ-positive/tau-negative and the Aβ-negative/tau-negative groups revealed only a nominal statistical difference in the entorhinal cortex and in the inferior frontal gyrus (*P* = 0.007 and *P* = 0.04, respectively).

Differences in longitudinal cortical MD between the Aβ-positive/tau-positive group and the Aβ-positive/tau-negative group were wide-spread and encompassed both the temporal and the parietal lobe as well as frontal regions [standardized-β range: 0.32–0.74; Fig. 1B(i)]. When including baseline cortical thickness of the same region the results remain highly consistent [standardized-β range: 0.27–0.64; Fig. 1B(ii); see Supplementary material and SupplementaryFig. 2A and B for the results of the analysis using the CT-corrected MD values].

**Figure 2:**
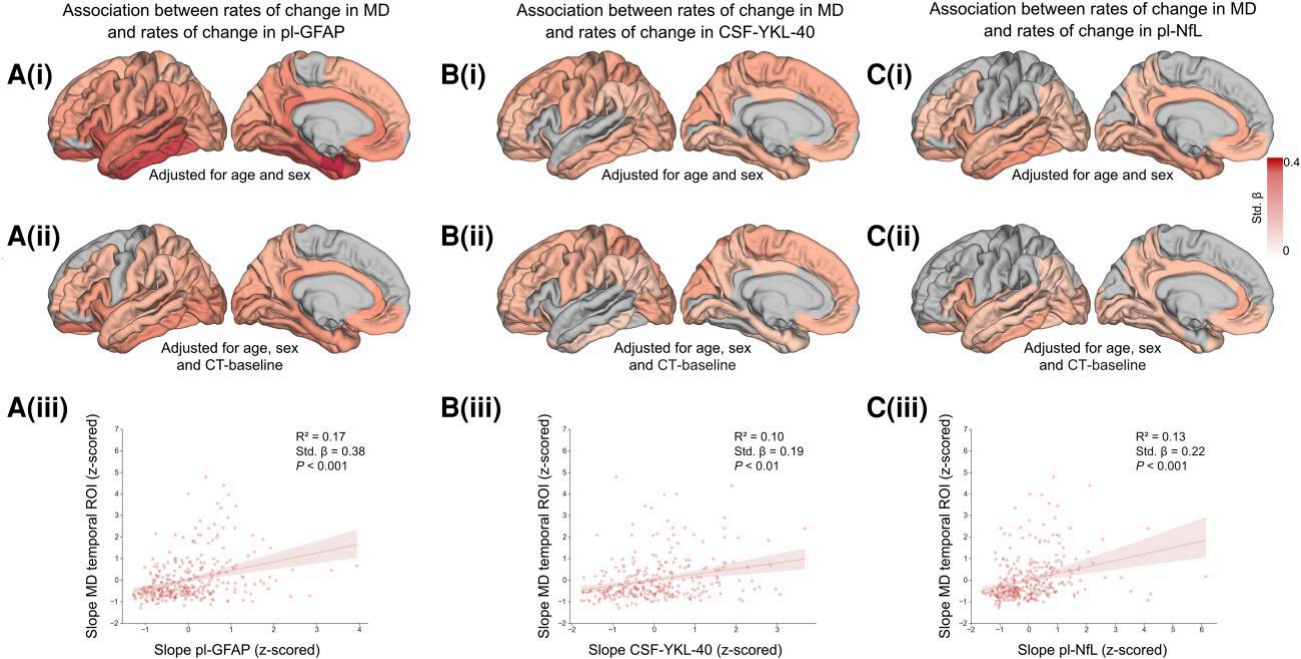
**Association between changes over time in mean diffusivity and changes over time in fluid markers of astrocytic activity and neurodegeneration**. The colour scale represents standardized-β values from the multiple regression. Only results corrected for multiple comparisons are displayed (false discovery rate, *P* < 0.05). Changes over time are quantified as individualized slopes from linear mixed-effect modelling. [**A**(**i**)] Association between changes over time in cortical mean diffusivity (MD) and changes over time in plasma levels of GFAP. [**A**(**ii**)] Association between changes over time in cortical MD and changes over time in plasma levels of GFAP including baseline cortical thickness from the same region as a covariate in the model. [**A**(**iii**)] Association between changes over time in cortical MD in the temporal region of interest (ROI) and changes over time in plasma levels of GFAP. [**B**(**i**)] Association between changes over time in cortical MD and changes over time in CSF levels of YKL-40. [**B**(**ii**)] Association between changes over time in cortical MD and changes over time in in CSF levels of YKL-40 including baseline cortical thickness from the same region as a covariate in the model. [**B**(**iii**)] Association between changes over time in cortical MD in the temporal ROI and changes over time in CSF levels of YKL-40. [**C**(**i**)] Association between changes over time in cortical MD and changes over time in plasma levels of NfL. [**C**(**ii**)] Association between changes over time in cortical MD and changes over time in in plasma levels of NfL including baseline cortical thickness from the same region as a covariate in the model. [**C**(**iii**)] Association between changes over time in cortical MD in the temporal ROI and changes over time in plasma levels of NfL. The standardized-β and *P*-value reported are derived from the multiple linear regression model described in the text. The shadowed areas around the regression line represent the 95% confidence interval.

The analysis focusing on *a priori* defined meta-ROIs, which could be employed to extract microstructural information from keys regions for Aβ or tau pathology, provided converging evidence showing significant differences in the trajectory of cortical MD between groups using both the early-Aβ and the temporal meta-ROI (all *P* ≤ 0.001; Fig. 1C and D and Supplementary Tables 1 and 2).

### Changes over time in fluid markers of astrocytic activity are associated with changes in cortical MD

In the subgroup of participants with available data on the astrocytic marker GFAP in plasma (*n* = 322), the regression analysis revealed a widespread positive association between longitudinal cortical MD and changes over time in levels of GFAP with higher standardized-β in temporal regions as well as in the posterior cingulate cortex [standardized-β range: 0.15–0.44; Fig. 2A(i)]. Including baseline cortical thickness in the same model did not significantly affect the overall results [standardized-β range: 0.14–0.26; Fig. 2A(ii); see Supplementary material and SupplementaryFig. 2C and D for the results of the analysis using the CT-corrected MD values]. Comparable results were found when testing CSF levels pf GFAP (Supplementary material and Supplementary Fig. 3). Similar results, although with lower standardize-β values, were also found when investigating the association with CSF level of astrocytic marker YKL-40 [*n* = 292; standardized-β range: 0.13–0.23, and including cortical thickness in the model, standardized-β range: 0.10–0.23; Fig. 2B(i) and (ii)]. The positive associations were found also when focusing on the meta-ROIs [GFAP—early-Aβ ROI: standardized-β = 0.32, *P* < 0.001; temporal ROI: standardized-β = 0.38, *P* < 0.001; YKL-40—early-Aβ ROI: standardized-β = 0.14, *P* < 0.05; temporal ROI: standardized-β = 0.19, *P* < 0.01; see Fig. 2A(iii) and B(iii) and Supplementary Fig. 4]. No significant association was found between changes over time in cortical MD and changes over time in CSF levels of the microglia-related marker s-TREM2 (Supplementary material).

### Changes over time in plasma NfL levels are associated with those in cortical MD

Longitudinal cortical MD was also associated with changes over time in plasma levels of neurodegeneration marker NfL with a widespread distribution [standardized-β range: 0.12–0.24; Fig. 2C(i)]. When including baseline cortical thickness in the model the positive association remain virtually unaffected in temporal and parietal regions but was no longer significant in several frontal areas [standardized-β range: 0.11–0.20; Fig. 2B(ii); see Supplementary material and SupplementaryFig. 2E for the results of the analysis using the CT-corrected MD values]. The positive association was replicated when focusing on the meta-ROIs [early-Aβ ROI: standardized-β = 0.20, *P* = 0.001; temporal ROI: standardized-β = 0.22, *P* < 0.001; Fig. 2C(iii)].

### Regional changes over time in cortical MD associated with worsening of cognitive performance

In the early-Aβ ROI, longitudinal cortical MD was positively associated with changes over time in both the TMT-B and the ADAS delayed recall score and negatively associate with changes in the mPACC score (TMT-B: standardized-β = 0.49, *P* < 0.001; ADAS delayed recall: standardized-β = 0.46, *P* < 0.001; mPACC: standardized-β = −0.49, *P* < 0.001) showing that steeper increase in MD over time were associated with steeper cognitive decline. Similar results were found when focusing on the temporal ROI (Table 2; see Supplementary Table 3 for the results of the analysis using the CT-corrected MD values).

**Table 2:** Associations between changes over time in mean diffusivity and changes over time in cognitive performance

	Standardized-β	Standard error	*t*-value	*P*-value	95% Confidence intervals
Early-Aβ ROI					
mPACC	−0.49	0.05	−10.19	<0.001	−0.59 to −0.40
TMT-B	0.49	0.08	6.44	<0.001	0.34 to 0.64
ADAS delayed recall	0.46	0.05	10.20	<0.001	0.37 to 0.55
Temporal ROI					
mPACC	−0.60	0.04	−13.61	<0.001	−0.69 to −0.52
TMT-B	0.61	0.08	7.98	<0.001	0.45 to 0.76
ADAS delayed recall	0.57	0.04	13.66	<0.001	0.49 to 0.65

Aβ = amyloid-β; ADAS = Alzheimer’s Disease Assessment Scale; mPACC = modified version of the Preclinical Alzheimer Cognitive Composite; ROI = region of interest; TMT-B = Trail Making Test Part B.

## Discussion

The present work showed that cortical MD, a metric derived by diffusion MRI, is able to track changes over time in the AD continuum. Cortical MD showed significantly different trajectories already in Aβ-positive participants who were still tau negative, and the differences were found consistently, both when testing across regions covering the entire neocortex and when focusing on *a priori* defined meta-ROIs. Furthermore, changes in cortical MD were associated with both changes in plasma levels of NfL, showing the sensitivity to the progression of neurodegeneration, and deterioration of cognitive performance, supporting the clinical relevance of the findings.

Of particular interest was the association between longitudinal changes in cortical MD and changes over time in peripheral markers of astrocytic activity namely plasma levels of GFAP and CSF levels of YKL-40. This result extends previous data showing a cross-sectional association between dMRI metrics and plasma level of GFAP^13^ and suggest cortical MD might reflect microstructural changes related to the neuroinflammatory response to AD pathology. The lack of correlation with changes over time in CSF levels of s-TREM2 further suggest a specific link between cortical MD and astrocytic activity. Multiple lines of research suggests that astrocytic dysfunction play a critical role in Alzheimer’s pathophysiology. For example, genetic data have revealed that a significant amount of the risk for sporadic AD is associated with genes that are primarily expressed in astrocytes.^31^ Transcriptomics studies have also confirmed that genes associated with the inflammatory response and, more specifically, with astrocytes are among the most reactive genes in the proximity of Aβ plaques.^32^ Moreover, reactive astrocytes have been found in the proximity of both Aβ plaques^33^ and neurofibrillary tangles^34^ in post-mortem studies. Although dMRI lack the biological specificity for targeting astrocytes, MD has been shown to correlates with astrocytosis, as measured with ^11^C-deuterium-L-deprenyl PET, in autosomal-dominant AD^14^ and a recent study on a rat model of neuroinflammation showed that dMRI metrics, including MD were sensitive to microstructural changes induced by astrocytic activity.^35^ In addition to that, an MRI post-mortem study have shown that a combination of dMRI and T2 relaxation was able to identify a signature of astrogliosis at least in blast-induced traumatic brain injury.^36^ In light of recent results of clinical trial showing that anti-Aβ therapies lower GFAP levels,^37^ the association between longitudinal cortical MD and changes over time in astrocytic markers appears especially relevant, suggesting dMRI might be able to track microstructural reorganization induced by treatment-related changes in the neuroinflammatory response. However, it is important to consider that other downstream events from protein accumulation could lead to an increase in MD. For example, synaptic degeneration would likely affect cortical microstructure resulting in a change in MD. Future studies should investigate the possible association between dMRI metrics and markers of synaptic disfunction. Moreover, although we found statistically significant differences in the trajectory of cortical MD already in Aβ-positive participants who were still tau negative and in regions typically associated with Aβ accumulation, the larger effects were observed in the Aβ-positive/tau-positive group and in the temporal ROI, which encompasses regions typically associated with tau accumulation. Aβ-positive/tau-positive participants are, by definition, more advance in the AD continuum; therefore, more robust results are expected, especially in areas encompassing regions particularly vulnerable to tau accumulation. However, this result serves as a reminder that we cannot pinpoint to a single underlining cause of microstructural changes with this study. Further work including both longitudinal Aβ- and tau-PET would help to elucidate to what extent changes over time in MD reflect events downstream of Aβ or tau accumulation or both. In addition, studying the regional association between microstructural changes and both Aβ and tau accumulation would help to clarify whether cortical MD can track or predict relevant pathological changes in the AD continuum. For example, a recent study has shown that cortical MD in the entorhinal cortex at baseline can predict tau accumulation especially in Aβ-positive individuals,^38^ showing the importance of considering synergetic effects of microstructural degeneration and both Aβ and tau accumulation. The mostly negative results in the comparison between the Aβ-negative/tau-negative group and the Aβ-positive/tau-negative group, when excluding participants who developed cognitive symptoms during the course of the follow-ups, could also suggest that Aβ accumulation alone cannot lead to microstructural changes. However, technical limitations could also have reduced our ability to detect subtle changes. The spatial resolution of clinically available dMRI sequence is still limited (2 × 2 × 2 mm^3^). Therefore, metrics of dMRI extracted from the cortical ribbon can be contaminated by partial-volume effects, reducing the specificity to cortical microstructure. This limitation could be overcome with specialized high-resolution dMRI approaches.^39^ Moreover, dMRI sequences including acquisitions with higher b-values would allow to implement dMRI models, e.g. mean apparent propagator (MAP)-MRI^40^ or neurite orientation dispersion and density imaging (NODDI),^41^ which have been shown to provide highly sensitive metrics to different aspects of the AD pathological process at least in cross-sectional studies.^13,42^ A recent study has also shown that metrics derived from MAP-MRI are more sensitive to age related differences compared to MD.^43^ However, it is important to notice that the type of data used in the present study, which still provide compelling results, can be easily acquired in the multicentre setup that characterizes clinical trials. Moreover, the sensitivity of cortical MD only to microstructural changes occurring when also cognitive decline take place could be considered as a strength of the metric, because cognitive decline is clearly the most relevant feature of the disease process. Future work should also investigate whether the surface-based approach, based on both a standard template and a standard atlas (i.e. the Desikan–Killiany atlas), represents the most accurate and robust processing steps. For example, a study-specific template could enhance the sensitivity to subtle changes, although it might risk the introduction of biases related to the choice of the MRI scans used to generate the template, especially in a possible multicentre clinical trial. Another limitation of the study is the lack of plasma and CSF samples for some participants. However, the subgroup analyses including fluid markers were still performed on groups of 322 and 292 participants for plasma and CSF, respectively, out of the total 424 participants.

With these limitations in mind, the current study, showing changes over time in cortical MD and associations with changes over time in fluid markers of astrocytic activity, supports a possible role of metrics derived from diffusion MRI in tracking early changes in the AD continuum. With many potential disease modifying therapies for AD reaching the advanced stages of clinical trials and the current lack of a convincing marker of neurodegeneration, metrics from dMRI emerge as promising measures to monitor the response to treatments during the later stages of clinical trials when target engagement has already been established. The next step would be to investigate whether disease-modifying treatments actually induce a normalization of dMRI metrics. The use of putative markers will become even more critical when clinical trials will move toward the preclinical stage of the disease where, by definition, clinical outcomes will become less sensitive and markers sensitive to small changes in the neurodegenerative process will be needed. In this context, metrics derived from dMRI by reflecting subtle cortical microstructural changes could become of pivotal importance.

## Supplementary Material

awad428_Supplementary_Data

## Data Availability

Anonymized data will be shared by request from a qualified academic investigator for the sole purpose of replicating procedures and results presented in the article and as long as data transfer is in agreement with EU legislation on the general data protection regulation and decisions by the Swedish Ethical Review Authority and Region Skåne, which should be regulated in a material transfer agreement.
