# Supplementary material for: Diffusion MRI tracks cortical microstructural changes during the early stages of Alzheimer’s disease
Source: Brain. 2023 Dec 21;147(3):961–9. doi: 10.1093/brain/awad428 (PMC10907088; doi:10.1093/brain/awad428)
Supplement: awad428_Supplementary_Data [file awad428_supplementary_data.pdf]

Supplementary materials

Supplementary methods

MRI processing

The dMRI series were processed following established steps based on freely available software. First, dMRI data were denoised using an *MRtrix3* routine which both estimate the noise level and denoise the data implementing an algorithm based on random matrix theory (see https://mrtrix.readthedocs.io/en/dev/dwi_preprocessing/denoising.html). After that, Gibbs ringing artifact were removed using the method proposed by Kellner et al.[1] implemented in *MRtrix3* (see, https://mrtrix.readthedocs.io/en/dev/reference/commands/mrdegibbs.html). Correction for movements artifact and eddy currents was implemented using *FSL eddy* (see, https://fsl.fmrib.ox.ac.uk/fsl/fslwiki/eddy). Data acquired with the inverse phase encoding direction were not available, therefore we were not able to implement an efficient and established strategy for correcting for susceptibility induced artifacts (e.g., *FSL topup*). To alleviate this problem, a non-linear registration procedure was employed when moving data from the dMRI space to the T1w space (target: T1w image, moving image: first volume from the dMRI series with b-value=0). The registration was implemented using *antsRegistration* (ANTs version: 2.1). *FSL* routines were used to fit the DTI model to the dMRI data. After that, the mean diffusivity (MD) map was warped to the space of the T1w scan acquired during the same session using the transformations derived from the non-linear registration and the MD map was projected on the cortical surface by sampling MD values at three equidistant points between white and pial surfaces starting and stopping within 25% from the borders of the cortical ribbon and averaging them to provide a single MD value for each vertex of the subject's cortical surface. These processing steps were performing using *FreeSurfer* commands as previously reported[2,3]. Finally, MD median values were extracted the 68 cortical regions of the Desikan-Killiany atlas. Critically for this study, the Desikan-Killiany atlas was warped into the individual time points T1w space following the *FreeSurfer* longitudinal pipeline which includes the creation of within subject templates as a first step of segmentation and reconstruction. Moreover, each time point is initialized within the template to reduce the variability in the optimization process (see supplementary figure 1 for an overview of the processing steps).

Cortical thickness-corrected changes over time in MD values

To account for changes over time in cortical thickness we corrected the regional mean diffusivity values for regional cortical thickness using the covariance method before estimating changes over time in mean diffusivity using LME. To this end, the regional MD values were first regressed against the corresponding cortical thickness (CT) values. After that, the CT-corrected MD values were obtained with: $CT\text{-}corrected\text{-}MD_{ROIp1} = MD_{ROIp1} - slope\text{-}model_{ROI} * (CT_{ROIp1} - CT_{ROImean})$, where MD_{ROIp1} = MD values of a specific *ROI* for the participant *p1*; $slope\text{-}model_{ROI}$ = slope of the model regressing MD values in a specific *ROI* against the cortical thickness values from the same ROI; CT_{ROIp1} = cortical thickness values of a specific *ROI* for the participant *p1*; $CT_{ROImean}$ = mean cortical thickness values of a specific *ROI* across all participants. The CT-corrected MD values were then used to compute CT-corrected estimate of MD changes over time by computing the individual-specific random slopes using the CT-corrected MD values as dependent variable on the LME models. Finally, we repeated the main analyses of the study employing the CT-corrected changes over time in MD instead of the uncorrected changes over time in MD.

Supplementary results

Regional changes over time in CT-corrected cortical MD differs between biomarker-defined groups

Longitudinal CT-corrected cortical MD still revealed microstructural differences, namely steeper increase in the CT-corrected MD over time, in the Aβ-positive/tau-negative group when compared with the control (Aβ-negative/tau-negative) group. Although the spatial extent of the results is more limited compared to the analysis without accounting for changes over time in CT, regions critically involved in the AD disease process (e.g., isthmuscingulate, the anterior portion of the cingulate cortex, the entorhinal cortex and the parahippocampal gyrus) still showed statistically significant differences in changes over time in MD (standardized-β range: 0.26 – 0.44; see supplementary figure 2A).

Difference in longitudinal CT-corrected cortical MD between the Aβ-positive/tau-positive group and the Aβ-positive/tau-negative group were still wide-spread and encompassed several regions in both the temporal and the parietal lobe as well as frontal regions (see supplementary figure 2B; standardized-β range: 0.24 – 0.64).

Changes over time in fluid markers of astrocytic activity are associated with changes over time in CT-corrected cortical MD

In the subgroup of participants with available plasma levels of GFAP (N=322), the regression analysis revealed a widespread positive association between longitudinal CT-corrected cortical MD and changes over time in levels of GFAP although the spatial extent was reduced in comparison to the analysis employing the uncorrected MD values (see supplementary figure 2B, standardized-β range: 0.16 – 0.29). Similar results, although with lower standardize-β values, were also found when investigating the association with CSF level of YKL-40 (N=292; see supplementary figure 2C, standardized-β range: 0.13 – 0.24).

Changes over time in plasma NfL levels are associated with changes over time in CT-corrected cortical MD

Longitudinal CT-corrected cortical MD was also associated with changes over time in plasma levels of NfL although with a reduction in the spatial extent of the results compared to the analysis using uncorrected estimate of changes over time in MD (see supplementary figure 2E, standardized-β range: 0.14 – 0.24).

Changes over time in CSF levels of sTREM-2 are not associated with changes over time in cortical MD

In the subgroup of participants with available longitudinal CSF sTREM-2 levels (N=292), the regression analysis revealed no significant association between changes over time in cortical MD and changes over time in sTREM-2. The analysis focusing on a-priori defined ROIs showed also no significant associations [early-Aβ ROI: standardized-β=0.07, $p>0.2$; temporal ROI: standardized-β=0.09, $p>0.1$]

Changes over time in CSF level of GFAP are associated with changes over time in cortical MD

In the subgroup of participants with available data on the astrocytic marker GFAP in CSF (N=292), the regression analysis revealed a widespread positive association between

longitudinal cortical MD and changes over time in levels of GFAP with higher standardized-β in temporal regions (see supplementary figure 1A, standardized-β range: 0.13 – 0.21). Including baseline cortical thickness in the same model did not significantly affect the overall results (see supplementary figure 1B, standardized-β range: 0.11 – 0.17). The positive associations were found also when focusing on the meta-ROIs [temporal ROI: standardized-β=0.21,p=0.001; early-Aβ ROI: standardized-β=0.17,p=0.005].

Supplementary references

1. Kellner E, Dhital B, Kiselev VG, Reisert M. Gibbs-ringing artifact removal based on local subvoxel-shifts. *Magn Reson Med*. 2016;76(5):1574-1581. doi:10.1002/mrm.26054
2. Montal V, Vilaplana E, Alcolea D, et al. Cortical microstructural changes along the Alzheimer's disease continuum. *Alzheimer's and Dementia*. 2018;14(3):340-351. doi:10.1016/j.jalz.2017.09.013
3. Rodriguez-Vieitez E, Montal V, Sepulcre J, et al. Association of cortical microstructure with amyloid-β and tau: impact on cognitive decline, neurodegeneration, and clinical progression in older adults. *Mol Psychiatry*. 2021;(September):1-10. doi:10.1038/s41380-021-01290-z

Supplementary tables

Supplementary table 1. Changes over time in mean diffusivity in a-priori defined ROI across biomarkers defined groups.

	Estimate	Standard error	t-value	P-value
**Early-Aβ ROI**				
Time*Group (A-T- – A+T-)	0.05	0.02	3.23	= 0.001
Time*Group (A-T- – A+T+)	0.14	0.02	8.27	< 0.001
Time*Group (A+T- – A+T+)	0.09	0.02	4.61	< 0.001
**Temporal ROI**				
Time*Group (A-T- – A+T-)	0.05	0.02	3.49	< 0.001
Time*Group (A-T- – A+T+)	0.19	0.02	11.56	< 0.001
Time*Group (A+T- – A+T+)	0.14	0.02	7.20	< 0.001

Fixed effects for the time (years) * biomarkers-defined group interaction from LME fitted in R version 4.2.2. A-T- = Aβ-negative/tau-negative, A+T- = Aβ-positive/tau-negative; A+T+ = Aβ-positive/tau-positive

Supplementary table 2. Median values of cortical MD ($\mu m^2/ms$) and cortical thickness (mm) in the a-priori defined ROIs

**Mean diffusivity**	Aβ-negative / tau-negative	Aβ-positive / tau-negative	Aβ-positive / tau-positive
Early-Aβ regions	0.957 (0.066)	0.977 (0.102)*	0.995 (0.081)*
Temporal ROI	0.883 (0.060)	0.911 (0.097)*	0.937 (0.094)*
**Cortical thickness**	Aβ-negative / tau-negative	Aβ-positive / tau-negative	Aβ-positive / tau-positive
Early-Aβ regions	2.34 (0.10)	2.30 (0.14)*	2.28 (0.13)*
Temporal ROI	2.61 (0.13)	2.57 (0.20)*	2.50 (0.18)*

Supplementary table 3. Associations between changes over time in mean diffusivity and changes over time in cognitive performance after accounting for changes over time in cortical thickness

Early-Aβ ROI					
	Standardized-β	Standard error	t-value	P-value	95% confidence intervals
mPACC	-0.35	0.05	-7.04	< 0.001	-0.45 – -0.25
TMT-B	0.30	0.08	4.04	< 0.001	0.15 – 0.44
ADAS delayed recall	0.32	0.05	6.8	< 0.001	0.23 – 0.41
Temporal ROI					
mPACC	-0.30	0.05	-5.92	< 0.001	-0.40 – -0.20
TMT-B	0.27	0.07	4.02	< 0.001	0.14 – 0.40
ADAS delayed recall	0.27	0.05	5.83	< 0.001	0.18 – 0.37

Abbreviations: mPACC: modified version of the preclinical Alzheimer cognitive composite; ADAS: Alzheimer’s Disease Assessment Scale; TMT-B: trail making test part B

Supplementary figures

Supplementary figure 1. Schematic representation of the main processing steps of MRI analysis

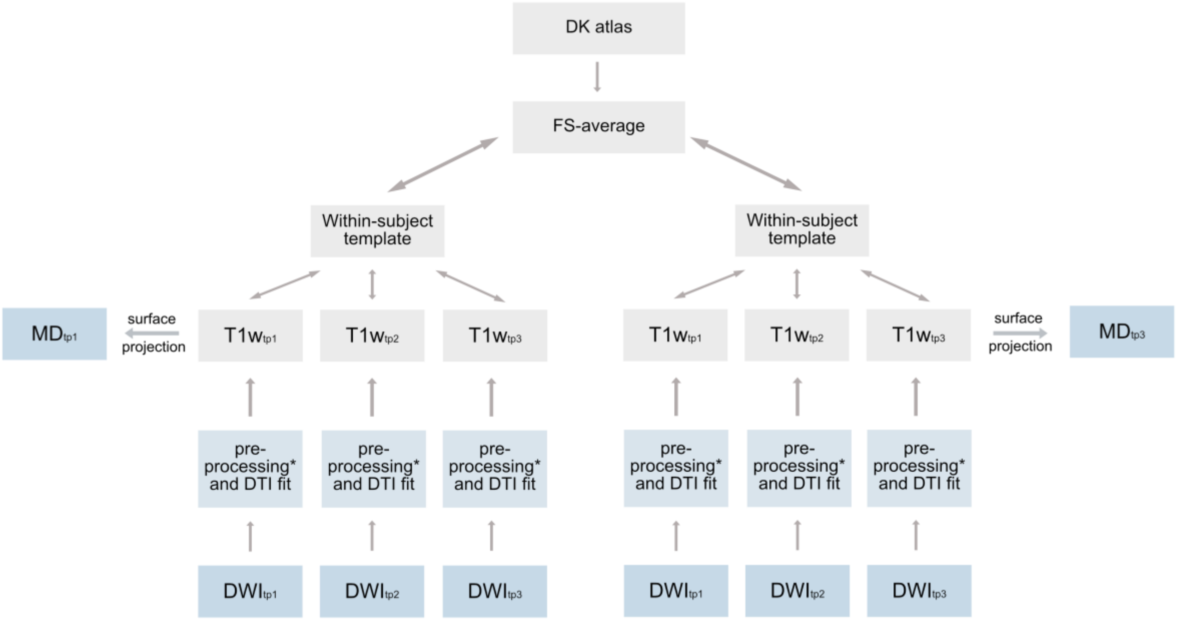

* Pre-processing steps of the dMRI series included: denoising, removal of Gibbs ringing, correction of movement-induced and eddy currents- induced artifacts, as described in the supplementary methods section. FS-average: common space defined by the *FreeSurfer* pipeline. DWI= diffusion weighted imaging, in this contest, it represents the raw dMIR series.

Supplementary figure 2. Changes over time in cortical mean diffusivity after subtracting the effect of changing over time in cortical thickness using the covariance method

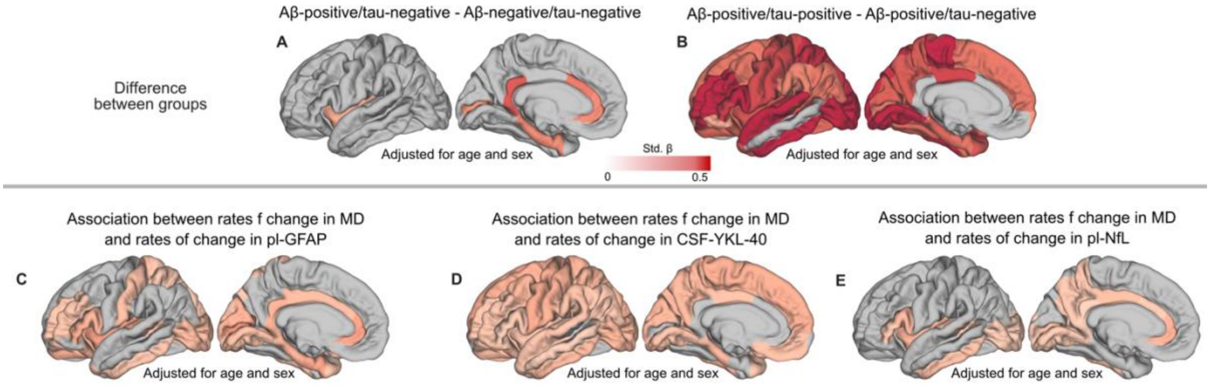

The color scale represents standardized β values from the multiple regression. Only results corrected for multiple comparisons are displayed (FDR, $p<0.05$). Changes over time are quantified as individualized slopes from LME modelling. The effect of changes over time in cortical thickness was accounted for using the covariace method before fitting the LME models. **A.** Difference in in cortical MD between Aβ-negative/tau-negative and Aβ-positive/tau-negative participants. **B.** Difference in changes over time in cortical MD between Aβ-positive/tau-negative and Aβ-positive/tau-positive participants. **C.** Association between changes over time in cortical MD and changes over time in plasma levels of GFAP. **D.** Association between changes over time in cortical MD and changes over time in CSF levels of YKL-40. **E.** Association between changes over time in cortical MD and changes over time in plasma levels of NfL. The standardized β and the p-value reported are derived from the multiple linear regression model described in the text. The shadowed areas around the regression line represent the 95% confidence interval.

Supplementary Figure 3. Association between changes over time in mean diffusivity and changes over time in CSF levels of GFAP

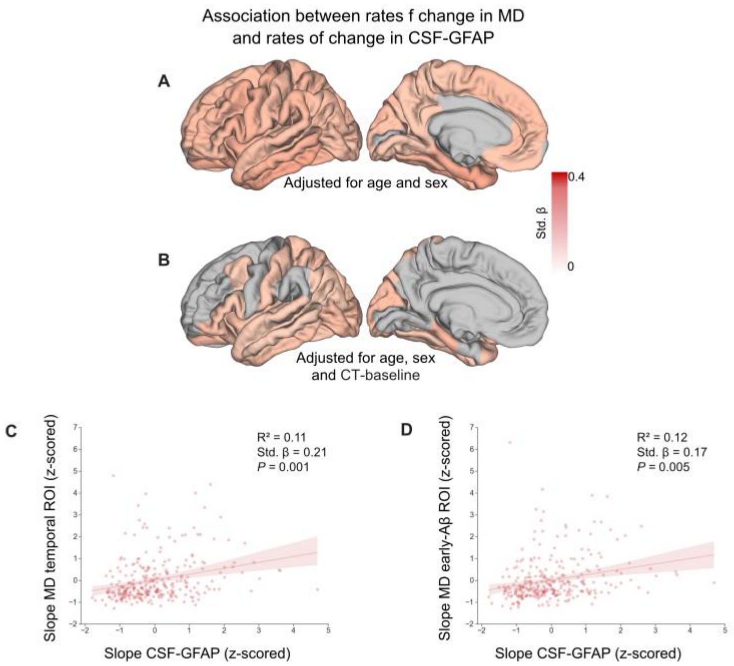

The color scale represents standardized β values from the multiple regression. Only results corrected for multiple comparisons are displayed (FDR, $p<0.05$). Changes over time are quantified as individualized slopes from LME modelling. **A.** Association between changes over time in cortical MD and changes over time in CSF levels of GFAP. **B.** Association between changes over time in cortical MD and changes over time in CSF levels of GFAP including baseline cortical thickness from the same region as a covariate in the model. **C.** Association between changes over time in cortical MD in the temporal ROI and changes over time in CSF levels of GFAP. **D.** Association between changes over time in cortical MD in the early-Aβ ROI and changes over time in CSF levels of GFAP. The standardized β and the p-value reported are derived from the multiple linear regression model described in the text. The shadowed areas around the regression line represent the 95% confidence interval.

Supplementary Figure 4. Association between changes over time in mean diffusivity in the Early-Aβ ROI and changes over time in flid markers of astrocytic activity and neurodegeneration

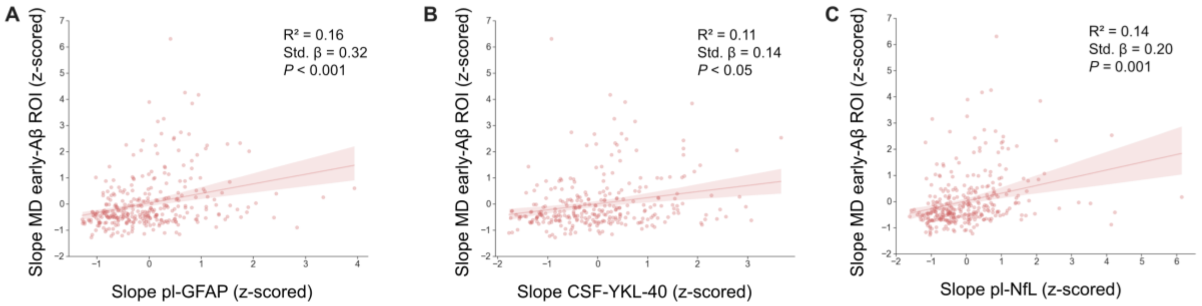

A. Association between changes over time in cortical MD in the early-Aβ ROI and changes over time in plasma levels of GFAP. **B.** Association between changes over time in cortical MD in the early-Aβ ROI and changes over time in CSF levels of YKL-40. **C.** Association between changes over time in cortical MD in the early-Aβ ROI and changes over time in plasma levels of NfL. The standardized β and the p-value reported are derived from the multiple linear regression model described in the text. The shadowed areas around the regression line represent the 95% confidence interval.
